# Supplementary material for: The MYC-YBX1 Circuit in Maintaining Stem-like Vincristine-Resistant Cells in Rhabdomyosarcoma
Source: Cancers (Basel). 2023 May 17;15(10):2788. doi: 10.3390/cancers15102788 (PMC10216535; doi:10.3390/cancers15102788)
Supplement: Supplementary file 1 [file cancers-15-02788-s001.zip › cancers-2360744-supplementary.pdf]

Figure S1

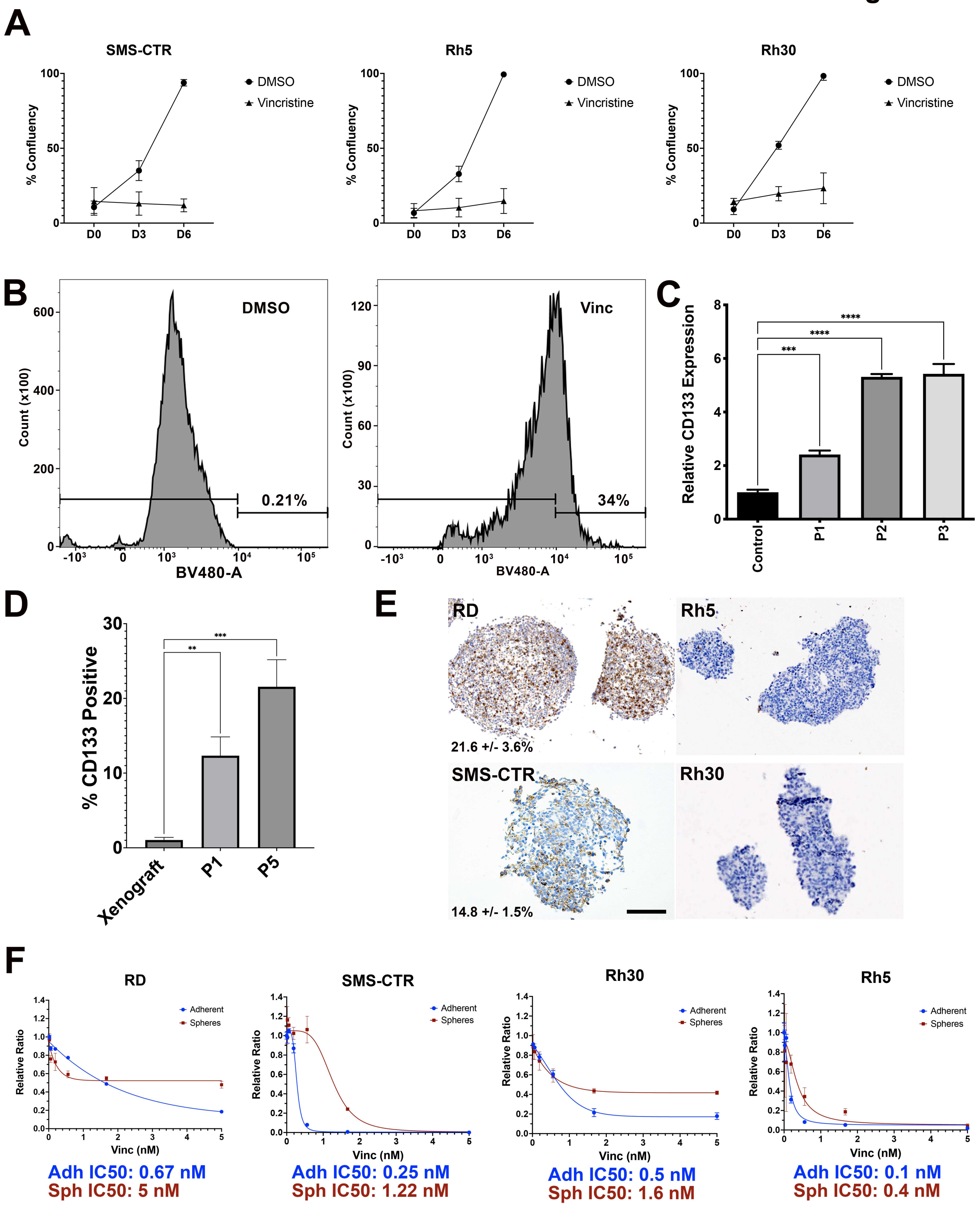

A
SMS-CTR
Rh5
Rh30
% Confluency
100
50
0
D0
D3
D6
DMSO
Vincristine
B
DMSO
Vinc
Count (x100)
BV480-A
0.21%
34%
C
Relative CD133 Expression
Control
P1
P2
P3
***
****
****
D
% CD133 Positive
Xenograft
P1
P5
**
***
E
RD
Rh5
SMS-CTR
Rh30
21.6 +/- 3.6%
14.8 +/- 1.5%
F
RD
SMS-CTR
Rh30
Rh5
Adherent
Spheres
Relative Ratio
Vinc (nM)
Adh IC50: 0.67 nM
Sph IC50: 5 nM
Adh IC50: 0.25 nM
Sph IC50: 1.22 nM
Adh IC50: 0.5 nM
Sph IC50: 1.6 nM
Adh IC50: 0.1 nM
Sph IC50: 0.4 nM

A

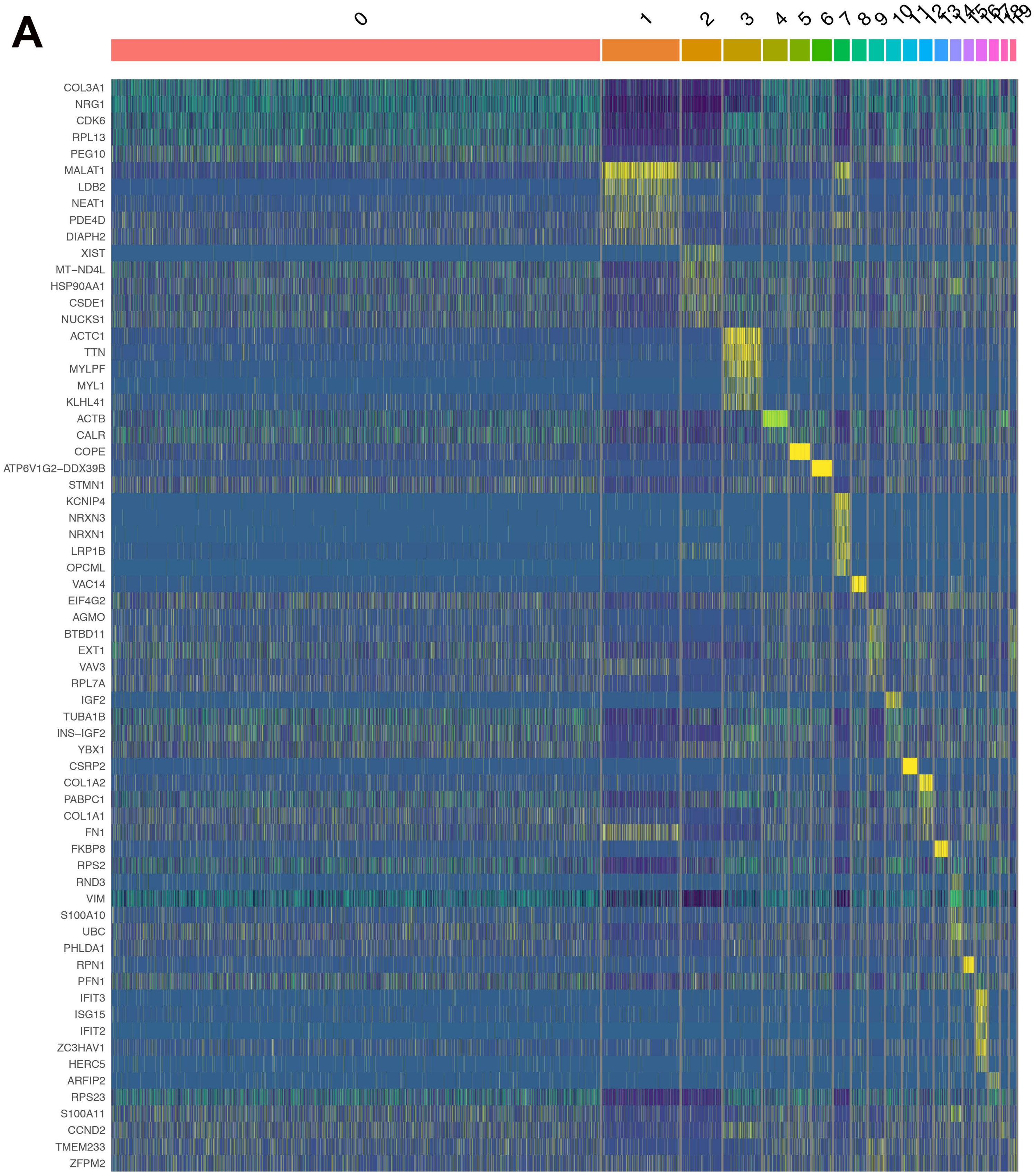
0
1
2
3
4
5
6
7
8
9
COL3A1
NRG1
CDK6
RPL13
PEG10
MALAT1
LDB2
NEAT1
PDE4D
DIAPH2
XIST
MT−ND4L
HSP90AA1
CSDE1
NUCKS1
ACTC1
TTN
MYLPF
MYL1
KLHL41
ACTB
CALR
COPE
ATP6V1G2−DDX39B
STMN1
KCNIP4
NRXN3
NRXN1
LRP1B
OPCML
VAC14
EIF4G2
AGMO
BTBD11
EXT1
VAV3
RPL7A
IGF2
TUBA1B
INS−IGF2
YBX1
CSRP2
COL1A2
PABPC1
COL1A1
FN1
FKBP8
RPS2
RND3
VIM
S100A10
UBC
PHLDA1
RPN1
PFN1
IFIT3
ISG15
IFIT2
ZC3HAV1
HERC5
ARFIP2
RPS23
S100A11
CCND2
TMEM233
ZFPM2

Figure S2

Figure S3

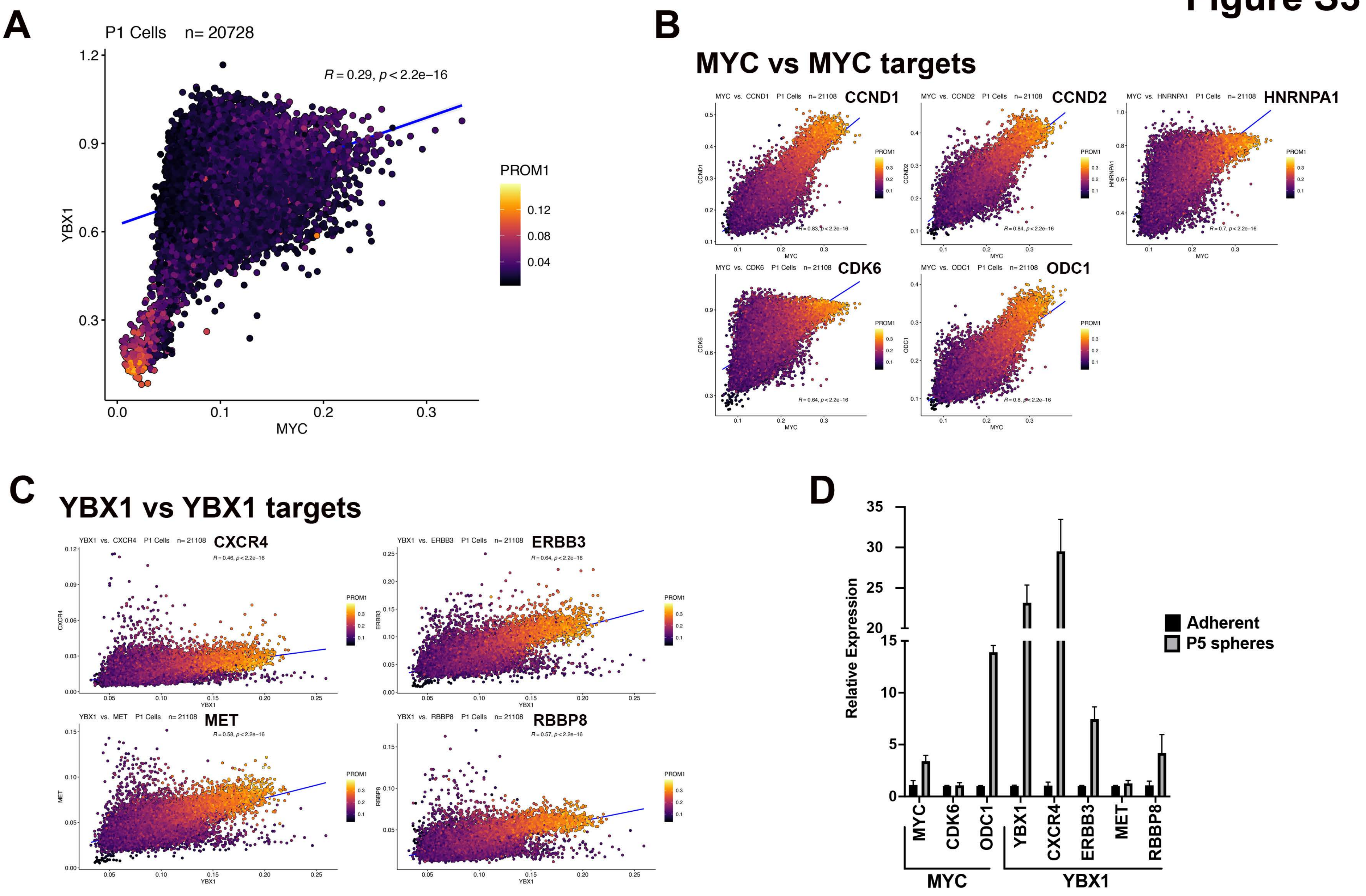

A
P1 Cells n= 20728
R = 0.29, p < 2.2e−16
YBX1
MYC
PROM1
B
MYC vs MYC targets
CCND1
CCND2
HNRNPA1
CDK6
ODC1
C
YBX1 vs YBX1 targets
CXCR4
ERBB3
MET
RBBP8
D
Relative Expression
MYC
CDK6
ODC1
YBX1
CXCR4
ERBB3
MET
RBBP8
Adherent
P5 spheres

Figure S4

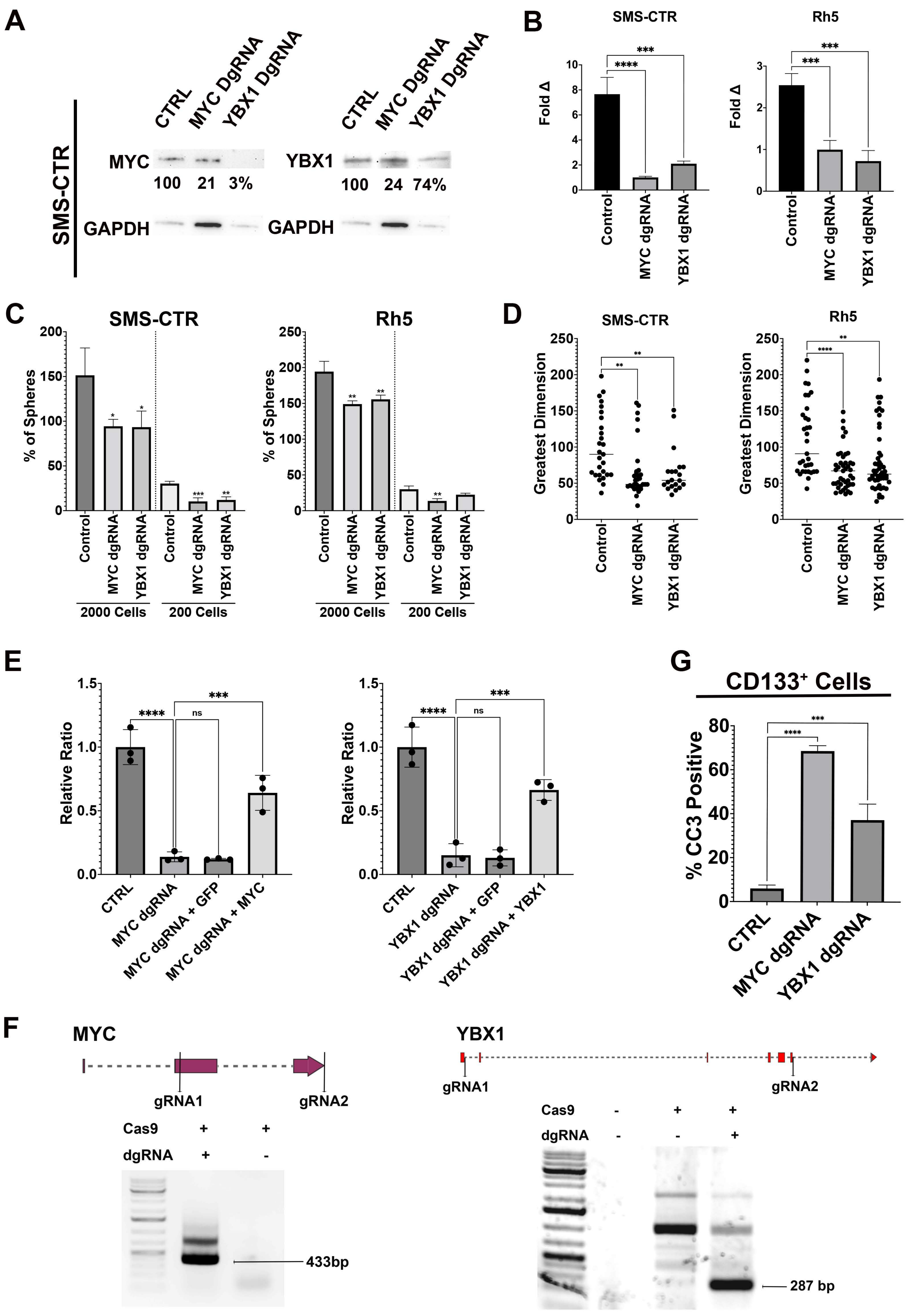

A
SMS-CTR
CTRL
MYC DgRNA
YBX1 DgRNA
MYC
100 21 3%
GAPDH
YBX1
100 24 74%
GAPDH
B
SMS-CTR
Rh5
Fold Δ
Control
MYC dgRNA
YBX1 dgRNA
C
SMS-CTR
Rh5
% of Spheres
2000 Cells
200 Cells
D
SMS-CTR
Rh5
Greatest Dimension
E
Relative Ratio
CTRL
MYC dgRNA
MYC dgRNA + GFP
MYC dgRNA + MYC
YBX1 dgRNA
YBX1 dgRNA + GFP
YBX1 dgRNA + YBX1
ns
G
CD133+ Cells
% CC3 Positive
F
MYC
gRNA1
gRNA2
Cas9
dgRNA
433bp
YBX1
287 bp

Figure S5

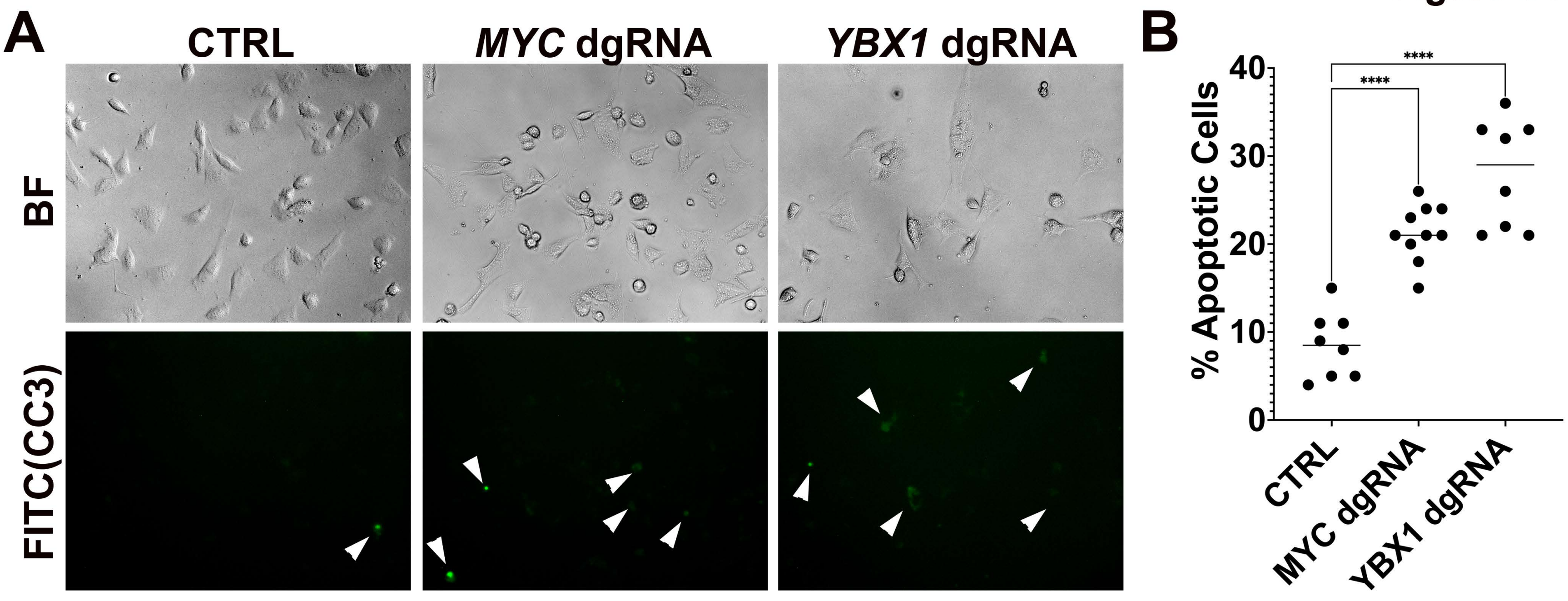
A
CTRL
MYC dgRNA
YBX1 dgRNA
BF
FITC(CC3)
B
% Apoptotic Cells
0
10
20
30
40
****
****
CTRL
MYC dgRNA
YBX1 dgRNA

Figure S6

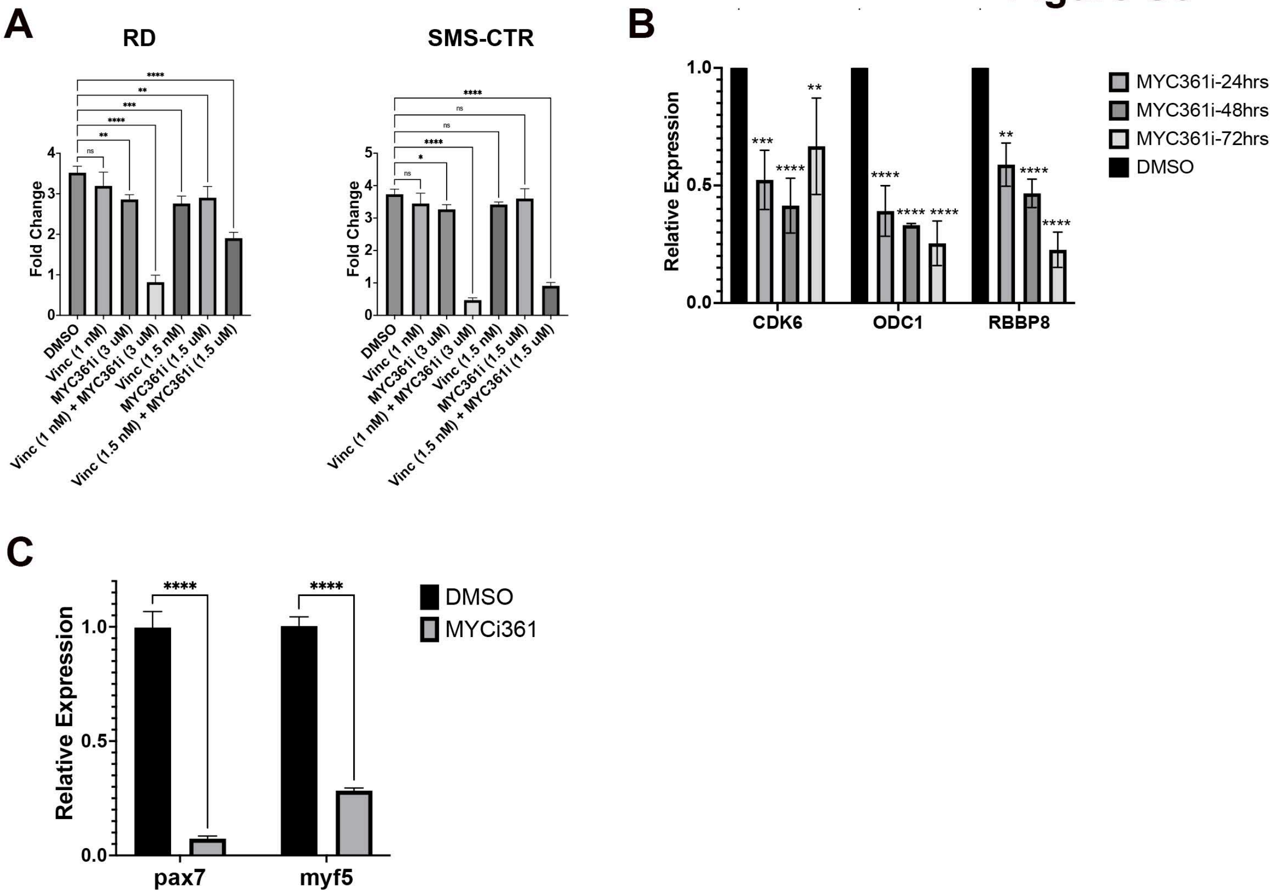
A
RD
SMS-CTR
Fold Change
DMSO
Vinc (1 nM)
MYC361i (3 uM)
Vinc (1 nM) + MYC361i (3 uM)
Vinc (1.5 nM)
MYC361i (1.5 uM)
Vinc (1.5 nM) + MYC361i (1.5 uM)
B
Relative Expression
CDK6
ODC1
RBBP8
MYC361i-24hrs
MYC361i-48hrs
MYC361i-72hrs
DMSO
C
Relative Expression
pax7
myf5
DMSO
MYCi361

A

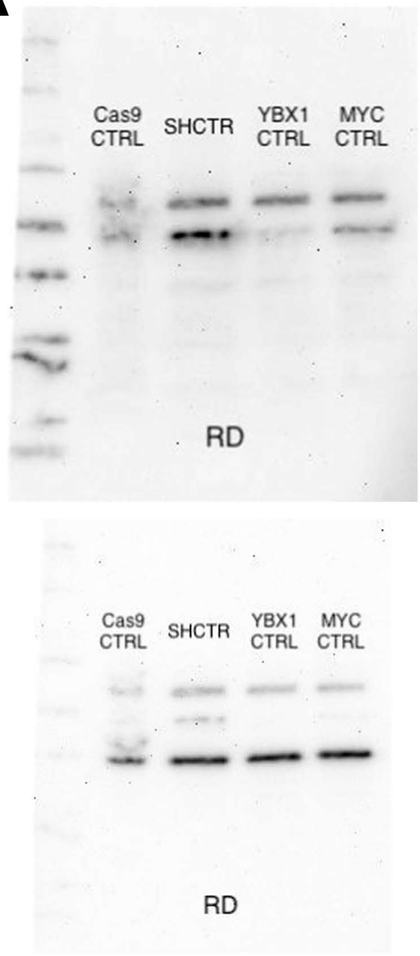

B

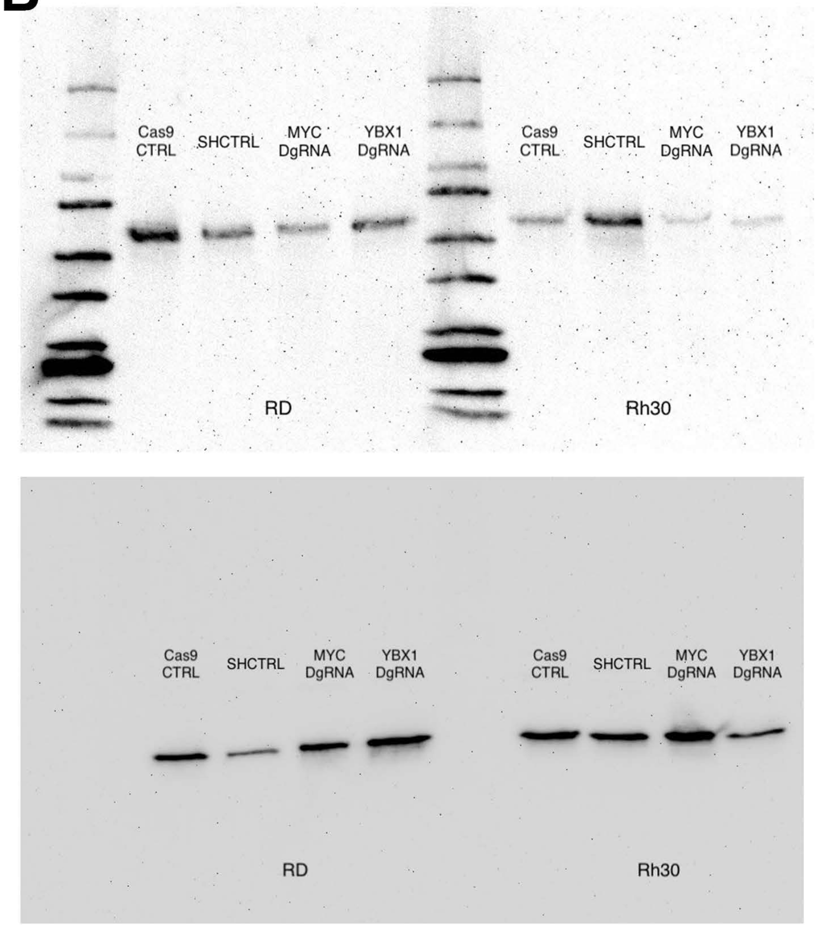

C

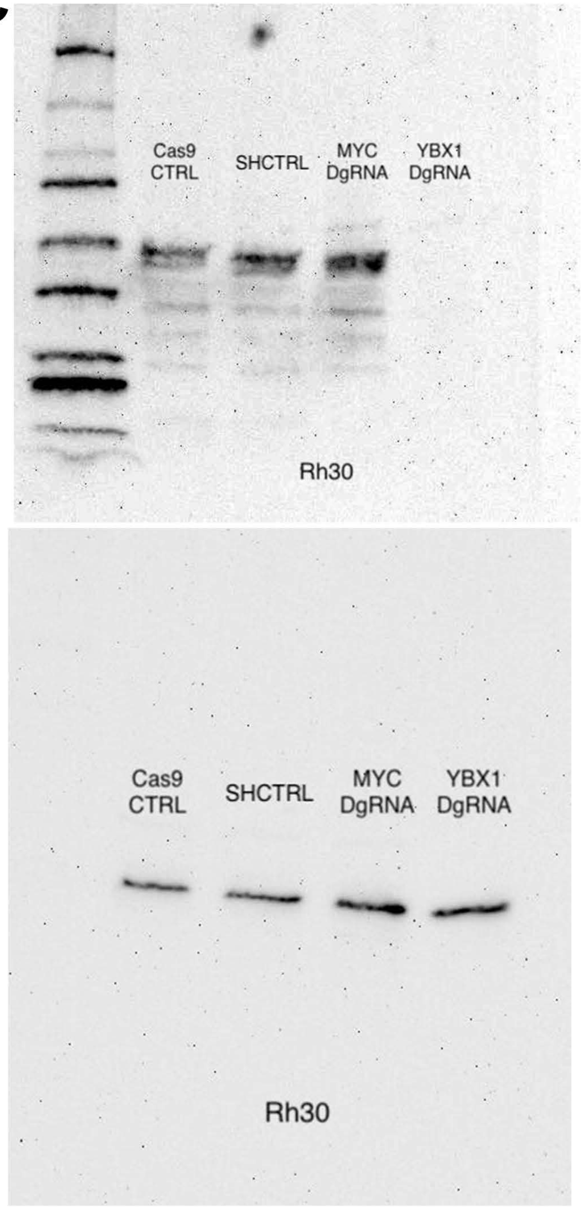

Figure S7

Table S1. Primers and gRNAs Used in the Study

RT-PCR PRIMERS		
**STEM GENES**	**FWD 5'-3'**	**REV 5'-3'**
CD133	ACTCCCATAAAGCTGGACCC	TCAATTTTGGATTCATATGCCTT
PAX7	TGTGACCGAAGCACTGTGCCC	AGCCGGTTCCCTTTGTCGCC
MYF5	CTGCCCAAGGTGGAGATCCTCA	CAGACAGGACTGTTACATTCGGGC
OCT4	GGTTCTCGATACTGGTTCGC	GTGGAGGAAGCTGACAACAA
NANOG	TCTGGACACTGGCTGAATCCT	CGCTGATTAGGCTCCAACCAT
SOX2	GTCATTTGCTGTGGGTGATG	AGAAAAACGAGGGAAATGGG
**MUSCLE GENES**	**FWD 5'-3'**	**REV 5'-3'**
MYOD	AGCACTACAGCGGCGACT	GCGACTCAGAAGGCACGTC
MYOG	CCTGCCGTGGGCGTGTAAGG	GGACTGCAGGAGGCGCTGTG
MYH8	GTGTTCACAGTCTTTCCGGC	CCCCACATCTTCTCCATCTC
CKM	CTCCTTCTCCGTCATGCTCT	GGTGGAGAAGCTCTCTGTGG
**YBX GENES**	**FWD 5'-3'**	**REV 5'-3'**
YBX1	AGGAGAAAAGGGTGCGG	ATGGTTACGGTCTGCTGCAT
CXCR4	GAAACCCTCAGCGTCTCAGT	AGTAGTGGGCTAAGGGCACA
ERBB3	TCCTTCCTGCAGTGGATTCG	ATCGTAGACCTGGGTCCCTC
MET	TGGGCACCGAAAGATAAACCT	CACTCCCCATTGCTCCTCTG
MYCN	AAGGCATCCTGGCTAGAGGA	GACCTCCAACACGGCTCTC
RBBP8	AGGGCGAAAGAGAAAAGCGA	TGGACAGGTCAAATACCGCC
**MYC GENES**	**FWD 5'-3'**	**REV 5'-3'**
MYC	CGGGTAGTGGAAAACCAGCCT	GAAGCTAAGGTTGAGGGGCA
CDK6	TCTGATTACTGCTCCGCGA	CAGAATCATTGCACTGAGGG
ODC1	CTGGGCGCTCTGAGATTGTC	CATCGAGGAAGTGGCAGTCA
SMAD3	TAATTTATTGCCGCCGCTCG	GGCCATCCAGGGACTCAAAC
ABCB1	AGGAAGCAACCAGATAAAAGAGAG	TGTGGCAAAGAGAGCGAAGC

RIP PRIMERS	FWD 5'-3'	REV 5'-3'
MYC	CGGGTAGTGGAAAACCAG	GAAGCTAACGTTGAGGGGCA
MYC	CGACGAGACCTTCATCAAAAAC	CGACGAGACCTTCATCAAAAAC
MYC	CAGCGACTCTG	GCTGGTGCATTTTCGGTTGT
MYC	AAACACAAACTTGAACAGCTAC	ATTTGAGGCAGTTTACATTATGG

gRNA	FWD 5'-3'	REV 5'-3'
MYC	CCAAGACCCCTTTAACTCAAGA	TTTCTTCCAGATATCCTCGCTG
MYC	AGGAGCAAAAGCTCATTTCTGA	GTCTCAAGACTCAGCCAAGGTT
YBX1	TTTCCATTGTCTTTTTCAGGGT	AAAAATGAGTCTGCAGGAGAGG
YBX1	CAGTCACCATCACCGCAA	AGCTCCGGCTAACGGTTC

Table S2: Quantification of Western blot densitometry

RD	MYC probe	Norm to GAPDH	Fold Δ CTRL		YBX1 probe	Norm to GAPDH	Fold Δ CTRL	
CTRL	5128.782	1.932926958			5858.397	1.069458034		
MYC dgRNA	3176.154	0.412937473	0.21363325	21.3633253	735.82	0.132357771	0.12376154	12.3761538
YBX1 dgRNA	6081.368	0.493646061	0.25538785	25.5387851	1742.497	0.310809075	0.29062297	29.0622975

	GAPDH
CTRL	2653.376
MYC dgRNA	7691.61
YBX1 dgRNA	12319.288

	GAPDH
CTRL	5477.912
MYC dgRNA	5559.326
YBX1 dgRNA	5606.326

Rh30	MYC probe	Norm to GAPDH	Fold Δ CTRL		YBX1 probe	Norm to GAPDH	Fold Δ CTRL	
CTRL	9085.752	1.023982884			11437.066	5.353970444		
MYC dgRNA	894.184	0.082467974	0.08053648	8.05364774	12175.51	3.213355492	0.60018178	60.0181776
YBX1 dgRNA	1177.598	0.259309938	0.25323659	25.3236595	10.536	0.003452674	0.00064488	0.0644881

	GAPDH
CTRL	8872.953
MYC dgRNA	10842.803
YBX1 dgRNA	4541.276

	GAPDH
CTRL	2136.184
MYC dgRNA	3789.033
YBX1 dgRNA	3051.548

SUPPLEMENTAL FIGURE LEGENDS

Figure S1. Vincristine treatment results in RMS resistant cells with stem-like characteristics. (A) Changes in the percentage of confluence over 6 days for Rh5, Rh30 and SMS-CTR treated with continuous exposure of vincristine (1 nM) or DMSO as quantified using the ImageJ software. Shown is the results from 3 independent experiments. (B) Representative flow cytometry plots with gates for unlabeled (left) and CD133 antibody-labeled RD cells (right). The percentage of CD133-positive cells is shown. (C) Quantitative RT-PCR results assessing *CD133* mRNA expression in serially passaged RD sphere cells compared to adherent parental cells (control). P = passage number. Each error bar represents standard deviation. (D) Quantitation results of immunohistochemistry for CD133 on xenograft tumor sections and serially passaged spheres generated from the RD line. Tissue cross-section images from 3 independent xenograft tumors and spheres were taken at 100x magnification, and positive-staining and total number of cells were quantified using the counter tool in the ImageJ software. The average percentage of CD133-positive cells in 3 representative cross sections from 3 independent xenograft tumors or spheres is shown for each cell line. One-way ANOVA, Dunnett's multiple comparisons test was performed in B and C. ** $P < 0.01$; *** $P < 0.001$; **** $P < 0.0001$. (E) Representative images of immunohistochemistry (IHC) for CD133 on RD xenograft tumor section, RD spheres, SMS-CTR spheres and Rh30 spheres. Average % positive cells with standard deviation is indicated. Rh5 and Rh30 spheres show no CD133 staining. (F) Dose-response curves of RD, SMS-CTR, Rh5 and Rh30 adherent cells and spheres treated with vincristine. The results were normalized to the vehicle control (DMSO). IC50 values for adherent and spheres are shown under each cell line.

Figure S2. Top differentially expressed genes in each cluster of RD sphere cells. (A) Heatmap showing top 5 differentially expressed genes in each cluster generated from UMAP analysis of RD sphere cells.

Figure S3. Correlation of MYC, YBX1 and their target genes in CD133-high expressing RMS cells. (A) Pearson correlation analysis on the relationship between *MYC* and *YBX1* expression in RD sphere cells from passage 1 (P1). Heatmap indicates expression levels of CD133 in RD cells analyzed. (B, C) Pearson correlation analysis on the relationship between MYC, YBX1 and their target genes. Heatmap indicates expression levels of CD133 in RD cells analyzed. (D) Quantitative RT-PCR analysis of *YBX*, *MYC* and their target genes in RD adherent cells and sphere cells at passage 5. Expression levels were normalized to adherent cells.

Figure S4. Targeted disruption of *MYC* or *YBX1* reduces RMS tumor cell growth and sphere formation. (A) Western blots against MYC and YBX1 in SMS-CTR cells with CRISPR/Cas9-mediated disruption of *MYC* and *YBX1*. DgRNA = double gRNA. Ratio of relative band intensity to the safe-harbor dgRNA sample is shown below each band. (B) Summary of CellTiter-Glo of SMS-CTR and Rh5 cells with CRISPR/Cas9-mediated disruption of *MYC* and *YBX1*. Luminescence intensity levels at day 5 were normalized to day 0. (C) Summary of limiting dilution sphere assays for SMS-CTR and Rh5 cells that were plated at 2000 and 200 cells. (D) Summary of sphere size (greatest dimension) quantification as determined using the Image J software. (E) Summary of rescue experiments using cell counts in stable Cas9-expressing RD line. Cells were first transduced with GFP, Cas9-resistant MYC and YBX1 lentiviral expression vector. Following at least 1 week of selection, cells were transduced with

CRISPR gene-specific targeting vectors. Following 3 days of antibiotic selection, cell counts were performed 5 days post plating. Cell counts were normalized to those of control cells transduced with safe harbor region dgRNA. (F) DNA electrophoresis demonstrating the deletion events in *MYC* and *YBX1* as amplified by the primers flanking the gRNAs (indicated by the arrows in the schematics above). Each band representing the deletion region is indicated by an arrow and expected size. (G) Quantitation of SMS-CTR with CD133:GFP knock-in stained with the NucView®530 Caspase-3 (CC3) substrate. For each group, 3 random images taken at 200x magnification were quantified for GFP-positive and CC3-positive (red channel) cells. Shown is the analysis from 3 replicate wells from 1 of 3 independent experiments. ANOVA Dunnett's multiple comparisons test: **** $P < 0.0001$, *** $P < 0.001$.

Figure S5. MYC and YBX1 are essential for maintaining viability of vincristine-resistant RMS cells. A. Representative images from live imaging using the fluorogeneic NucView®488 Caspase-3 substrate for detecting apoptotic cells in SMS-CTR DTPs**.** Transduced cells following antibiotics selection for 3 days were imaged 8 hours after restarting vincristine (1.5 nM) treatment, and 1 hour of exposure to the substrate. (B) Quantitative summary of the results from the imaging study for apoptosis. For each condition, images were taken from at least 8-10 random fields at 200x magnification across 3 biological replicates.

Figure S6. MYCi361 and vincristine synergize in inhibiting tumor cell growth. (A) Summary of representative CellTiter-glo viability assays in RD and SMS-CTR cells treated with vincristine, MYCi361 and the combination of vincristine and MYCi361. For the combination therapy, cells were treated first with vincristine for 3 days followed by MYCi361 for 3 days.

Error bars represent standard deviation of 3 replicate wells for each condition. (B) Quantitative RT-PCR assessing expression levels of *MYC* target genes in RD cells treated with DMSO and MYCi361 (3 mM). ns = not significant; * = $P < 0.05$; ** = $P < 0.01$; *** = $P < 0.005$; **** = $P < 0.0005$ by two-way ANOVA analysis with Dunnett's multiple comparisons test. (C) Quantitative RT-PCR in representative zebrafish RMS tumors treated with DMSO (vehicle control) and MYCi361 (100 mg/kg), n = 3 each. Tumors were harvested 4 days post-treatment. Error bars represent standard error of means.

Figure 7. Original raw images of Western blots. (A) RD cells probed with YBX1 antibody. (B) RD and Rh30 cells probed with MYC antibody. (C) Rh30 cells probed with YBX1 antibody.

SUPPLEMENTAL METHODS:

Single Cell RNA Sequencing Sample and Data Processing:

For single cell RNA sequencing dta pre-processing, base calls are converted to fastq format using Illumina's bcl2fastq and then demultiplexed using by a custom script called make_sample_fastqs.py. Demultiplexed reads are then adaptor clipped using trim_galore with default settings. Trimmed reads are mapped to the 3'UTR extended reference STAR index generated from ensembl annotations for macaque. Uniquely mapping reads are extracted, and duplicates are removed using the unique molecular identifier (UMI) sequence, reverse transcription (RT) index, and read 2 end-coordinate (i.e. reads with identical UMI, RT index, and tagmentation site will be considered duplicates). To generate expression matrices, the number of UMIs for each cell mapping to the exonic and intronic regions of each gene are calculated. For multi-mapped reads, reads are assigned to the closest gene, except in cases where another intersected gene falls within 100 bp to the end of the closest gene, in which case the read is

discarded. For most analyses we include both expected-strand intronic and exonic UMIs in per-gene single-cell expression matrices

Cell-based Assays:

RMS cell growth was quantified by direct cell counting or the ATP-based Cell Titer Glo luminescent cell viability assay (Promega, Madison, WI). For cell counts, RMS cells were transduced with lentivirus for 1-2 days and selected with the appropriate antibiotic for 3 days prior to plating. Cell counts were performed at 5-7 days post plating at a starting density of 10,000-20,000 cells per well in 24-well plates. For cell growth rescue experiments using the stable line harboring the Cas9 expressing cassette and Cas9-resistant GFP, Cas9-resistant MYC or YBX1, cells were transduced first with lentivirus for gene-specific dgRNAs for 1-2 days. Following antibiotic selection for 3 days, cells were plated for cell counts for the Cell Titer Glo assay. Cell counts were performed 5-6 days post-plating. For the Cell Titer Glo assay, cells were plated in 96-well plates at 1000-2000 cells per well, and the luminescence was read using a microplate reader 6 days post-plating (BioTek Synergy H1, Winooski, VT).

Spheres (“rhabdospheres”) were induced in stem cell (neurobasal) medium enriched with growth factors (EGF, bFGF, PDGF-A and PDGF-B) as previously described (39). For flow cytometry-based Annexin V assay, transduced RD cells 3 days-post antibiotic selection were incubated in the stem cell medium in the 10-cm low-attachment plates for 5 days prior to harvesting for the assay.

Immunohistochemistry (IHC) and Immunofluorescence (IF)

A tissue microarray created from archived paraffin tissue blocks of human RMS tumor samples was obtained from Seattle Children's Hospital. Immunohistochemistry was performed at the Histology and Imaging core facility at University of Washington. The following antibodies including dilutions were used for IHC: rabbit polyclonal anti human mouse monoclonal anti-Ki-67, (1:100, clone MIB1, Dako, Santa Clara, CA), rabbit monoclonal anti-MYC (1:100, clone E5Q6W; Cell Signaling, Danvers, MA), rabbit polyclonal anti-YBX1(1:50; clone D299; Cell Signaling, Danvers, MA) and rabbit monoclonal anti-CD133 (Prominin) (1:100; D2V8Q; Cell Signaling, Danvers, MA). The following antibodies including dilutions were used for IF: rabbit monoclonal anti-MYC (1:150, clone E5Q6W; Cell Signaling, Danvers, MA), rabbit polyclonal anti-YBX1(1:150; clone D299; Cell Signaling, Danvers, MA) and rabbit monoclonal anti-CD133 (Prominin) (1:150; D2V8Q; Cell Signaling, Danvers, MA).

Real-time Apoptosis Detection on cultured RMS cells

For detecting apoptosis in cultured cells, adherent RMS cells or sphere cells were incubated with NucView® 530 or 488 Caspase 3 substrate (Biotium, Fremont, CA), diluted to 2 μM in 500 μL medium, for 1 hour prior to imaging by microscopy using the Cy3 channel.

Western Blots

Human cell line and zebrafish tumor whole cell lysates prepared in RIPA with protease inhibitors and 2x sample buffer were electrophoresed on a 4-15% gradient SDS-polyacrylamide gel (Bio-Rad, Hercules, CA) and transferred to PVDF membranes using the TurboTrans-Blot (Bio-Rad, Hercules, CA). Blots were blocked in 5% milk-TBST and probed using the following antibodies and dilutions: GAPDH (1:2000; Cell Signaling, Danvers, MA); rabbit monoclonal

anti-MYC (1:1000, clone E5Q6W; Cell Signaling, Danvers, MA), rabbit polyclonal anti-YBX1(1:1000; clone D299; Cell Signaling, Danvers, MA); rabbit polycloncal anti-phospho-c-Myc (Ser81) (1;1000; MilliporeSigma). Goat anti-mouse or anti-rabbit HRP conjugated IgG secondary antibodies were obtained from Santa Cruz Biotechnology.

Quantitative RT-PCR

Human or zebrafish cells were lysed in TRIzol reagent (Thermo Fisher Scientific, Waltham, MA) and RNA was isolated per manufacturer's protocol. Approximately 1 microgram of RNA was used for cDNA synthesis using the High Capacity cDNA Reverse Transcription kit (Thermo Fisher Scientific, Waltham, MA). SYBR Green-based quantitative PCR was subsequently performed using gene-specific primers (Table S1) in a light cycler (CFX Connect Real-time PCR Detection System) from Bio-Rad (Hercules, CA).
